# Inferior Vena Cava Agenesis: An Underrated Cause of Deep Venous Thrombosis

**DOI:** 10.7759/cureus.33667

**Published:** 2023-01-11

**Authors:** Muthumeena Kannappan, Sruthilatha Sakthi Velavan

**Affiliations:** 1 Hospital Medicine/Primary Care & Public Health, Franciscan Health, Lafayette, USA; 2 Biology, Indiana University, Bloomington, USA

**Keywords:** deep vein thrombosis (dvt), ivc embryology, collateral veins, agenesis, inferior vena cava

## Abstract

The absence of the inferior vena cava (IVC) is a rare abnormality reported in less than 1% of the population. The condition is usually the result of defects during embryogenesis. The collateral veins are enlarged with agenesis IVC, enabling blood transport to the superior vena cava. Although the alternate pathways enable venous drainage of the lower extremities, IVC agenesis (IVCA) may predispose to venous hypertension and complications, including thromboembolism. This report includes a case of a 35-year-old obese male who presented with deep vein thrombosis (DVT) in his left lower extremity (LLE) with no predisposing factors, which led to an incidental discovery of the inferior vena cava agenesis. Imaging showed thrombosis of the deep veins of the LLE, absence of the IVC, enlarged paralumbar veins, filling of the superior vena cava, and left renal atrophy.

The patient responded to therapeutic heparin infusion, and catheter placement and thrombectomy were performed. The patient was discharged on the third day with medications and vascular follow-up. It is essential to recognize the complications of IVCA and its correlation with other findings, such as atrophy of the kidney. The agenesis of IVC is a highly under-recognized cause of DVT of the lower extremities in the young population without other risk factors. Hence, a complete diagnostic evaluation is necessary for this age group, including imaging for vascular anomalies, besides the thrombophilic screening.

## Introduction

The inferior vena cava (IVC) is a retroperitoneal vessel formed by the union of the right and left common iliac veins. The IVC is on the right of the midline, formed at the fifth lumbar vertebrate (L5) level, and carries deoxygenated blood from the lower extremities, pelvis, and abdomen to the right atrium of the heart [1]. The agenesis of IVC is the complete absence of the vessel, and this condition is reported only in 0.0005% to 1% of the general population [2]. The cause of inferior vena cava agenesis (IVCA) is related to embryological dysgenesis, although rarely due to perinatal thrombosis [3]. Patients with IVCA are usually asymptomatic but may present with lower back and abdominal pain [4]. In the absence of IVC, venous blood from its drainage area uses an alternate pathway to reach the right atrium. The alternative route in the absence of an IVC is the azygos vein which drains the lower parts of the body to terminate in the superior vena cava [5]. However, alteration could predispose to clinical conditions such as deep vein thrombosis (DVT) [3]. The prevalence rate of DVT is 1 in 1000 in the general population and is associated with high morbidity and mortality [4]. The occurrence of DVT in patients less than 30 years old is uncommon, and approximately 5% of them are found to have IVCA [6].

## Case presentation

A 35-year-old obese American male without any significant past medical history presented to the emergency for sudden onset of the left leg “Charley horse” with subsequent left calf tightness, pain, and swelling of one-day duration. He noticed an increasing purple discoloration of his left leg throughout the day. He was a non-smoker and denied any history of recent travel, extended immobility, trauma, surgery, cancer, or prior history of venous thromboembolism. His family history was positive for unprovoked pulmonary embolism in his father without any evidence of genetic predisposition. On examination, his entire left lower extremity (LLE) was significantly swollen with purplish discoloration and tenderness to touch with delayed capillary refill and intact distal pulses, sensations, and motor function. Laboratory findings, including the hypercoagulation panel, were unremarkable. The electrocardiogram showed sinus tachycardia, and the chest X-ray was unremarkable. The ultrasound venous duplex of the lower extremity showed near-total obstruction of the left deep venous system with thrombosis from the common femoral vein through the popliteal veins with a small amount of flow seen within the distal left saphenous vein. The patient was hospitalized for acute, unprovoked, and symptomatic DVT of the LLE and started on heparin infusion with a plan for percutaneous thrombectomy. 

**Figure 1:**
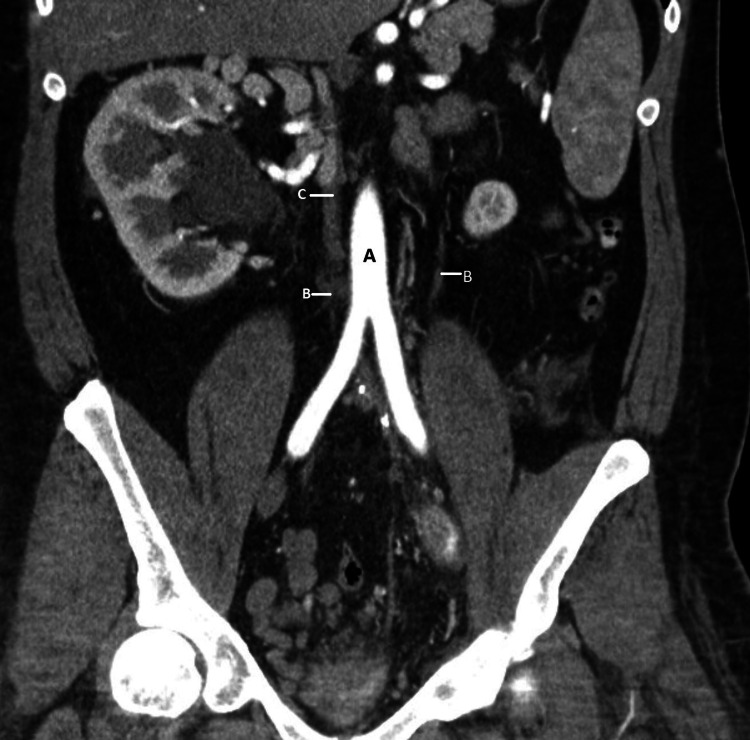
Contrast tomography of abdomen and pelvis A: Abdominal aorta; B: Absence of inferior vena cava, enlarged paralumbar veins; C: Azygos vein

**Figure 2:**
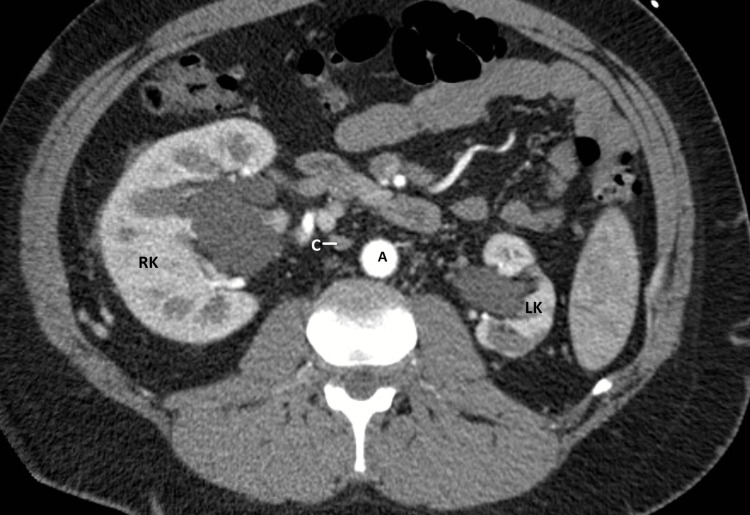
Contrast tomography of abdomen and pelvis A: Abdominal aorta; RK: Absence of inferior vena cava, enlarged right kidney; LK: Atrophic left kidney; C: Azygos vein

A venogram of the LLE showed a thrombus extending from the popliteal access cranially through the iliac veins without a clear path to the IVC. Numerous attempts to pass a guidewire into the IVC were unsuccessful, and the procedure was terminated. Contrast tomography of the abdomen and pelvis (CTAP) was done to evaluate the baseline abdominal and pelvic venous anatomy. It showed an absence of the IVC with abdominal pelvic venous outflow through a well-performed paralumbar, retroperitoneal body wall collaterals, and ultimate opacification of the superior vena cava (SVC) (Figure 1). Additional left renal atrophy with compensatory hypertrophy of the right kidney was noted (Figure 2).

The patient was continued on therapeutic heparin dose with a view to lifelong anticoagulation pending vascular recommendations. The vascular surgeon performed an angiogram remarkable for extensive DVT involving the left popliteal vein, femoral vein, and iliac vein as well; IVC could not be visualized due to agenesis. Through ultrasound-guided left popliteal vein access, a thrombolytic catheter was placed in the left iliac, femoral, and common femoral veins, and balloon angioplasty of the iliac venous system, including external and common iliac veins, and common femoral and femoral vein was performed. On the second day, after catheter placement, reimaging showed clearing of the thrombus in the femoral, common femoral, and iliac veins, with some persistent thrombus in the external iliac, common iliac, and common femoral veins, which were treated by performing thrombectomy and removal. On the third day, the patient was taken back to the interventional lab to follow up on the lytic process, and imaging showed significant clearing of thrombus with some residual clots noted in the iliac and internal iliac veins that were removed by thrombectomy. Completion angiogram revealed significantly improved outflow via the internal iliac vein and the venous plexus. The thrombolytic catheter was removed on the third day, and the patient was discharged on a full dose of enoxaparin sodium and clopidogrel with vascular follow-up. 

## Discussion

The agenesis of the IVC is rarely reported in the literature, accounting for only 0.005% to 1% of the population [7]. Since this anatomical variation is primarily asymptomatic, it is less frequently reported in healthy and younger individuals [8]. The diagnosis of IVCA is often incidental during abdominal surgery, cadaveric dissection, or radiological procedures. When a CTAP scan was done in a case of intra-abdominal cancer, the IVCA was detected, and various other enlarged venous pathways that compensated for the missing IVC were found [9]. Five instances of IVCA were detected during electrophysiological procedures [7]. Some tests that are useful for finding and diagnosing IVCA are ultrasound, CT scan, MRI, or venogram of the abdomen [10]. 

Typically the IVC is formed at the L5 level by the union of the right and left common iliac veins [11]. Besides the right adrenal gland, right gonad, and liver, four lumbar veins from the posterior abdominal wall also drain into the IVC [11]. The paralumbar veins interconnect the lumbar and the ascending lumbar veins [11]. The azygos venous system is on both sides of the vertebral column, consisting of the azygos vein and its tributaries, hemiazygos, and accessory hemiazygos vein [11]. The azygos vein extends superiorly from the right ascending lumbar vein and drains into the superior vena cava [12]. The hemiazygos vein begins as the left ascending lumbar vein and crosses the midline at the eighth thoracic vertebra (T8) level to join the azygos vein [12]. Lastly, the accessory hemiazygos vein begins in the posterior mediastinum and descends to cross the aorta at T7 to join the azygos vein [11]. The azygos venous system interconnects the inferior and superior vena cava [12]. Besides the azygos system, the superficial epigastric veins-thoracoabdominal veins-subclavian veins, and the inferior epigastric veins-superior epigastric veins-subclavian veins also serve as collateral pathways of venous drainage connecting the upper and lower parts of the body [13]. All three paths may enlarge and serve as alternate drainage when one of the large veins is blocked. 

**Figure 3:**
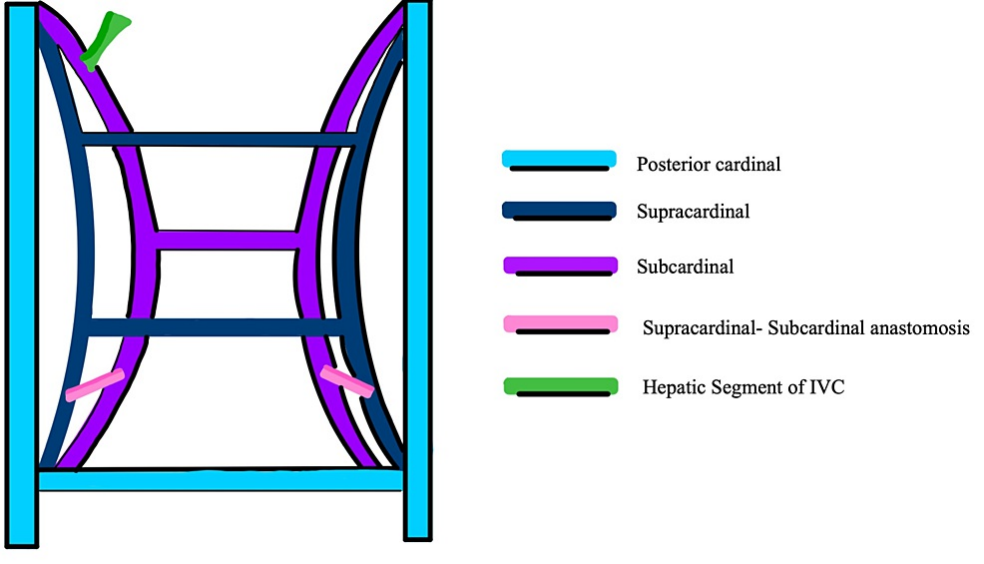
Embryonic veins of the abdomen and pelvis

**Figure 4:**
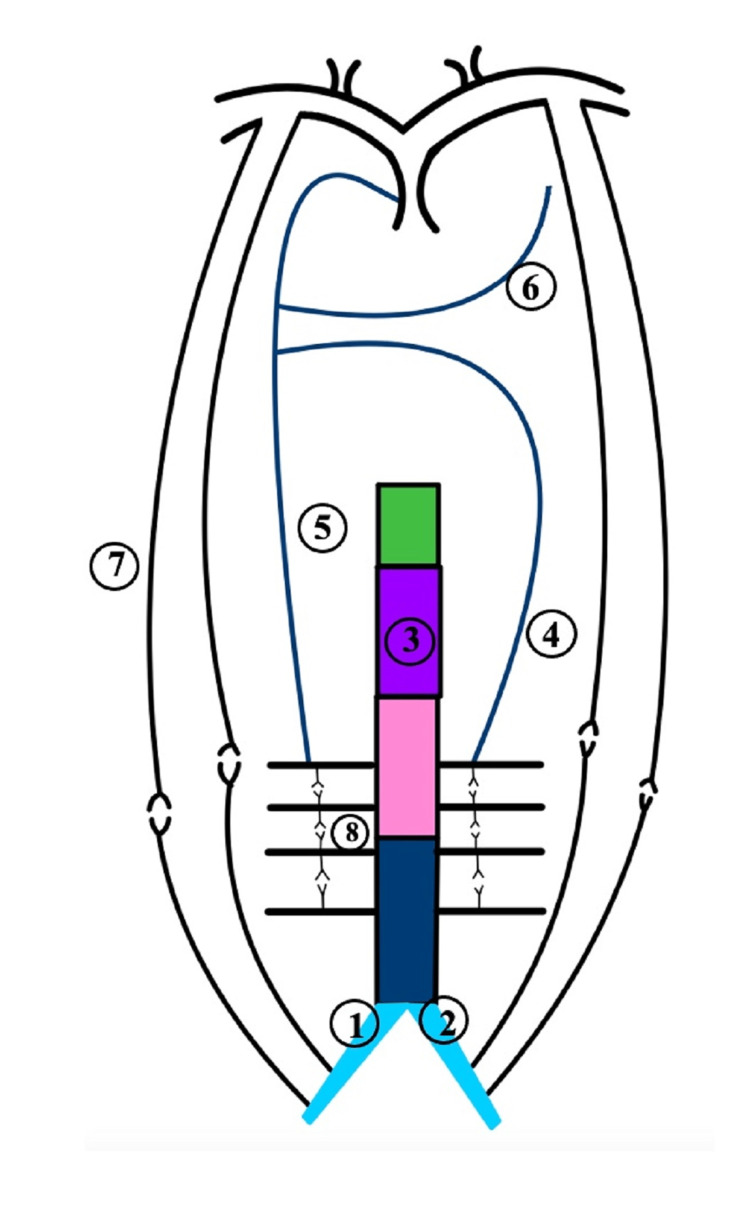
Embryogenesis of the inferior vena cava and azygos venous system Right common iliac vein (1); left common iliac vein (2); inferior vena cava (3); hemiazygos vein (4); azygos vein (5); accessory hemiazygos vein (6); abdominal wall collaterals (7); lumbar and paralumbar veins (8)

**Figure 5:**
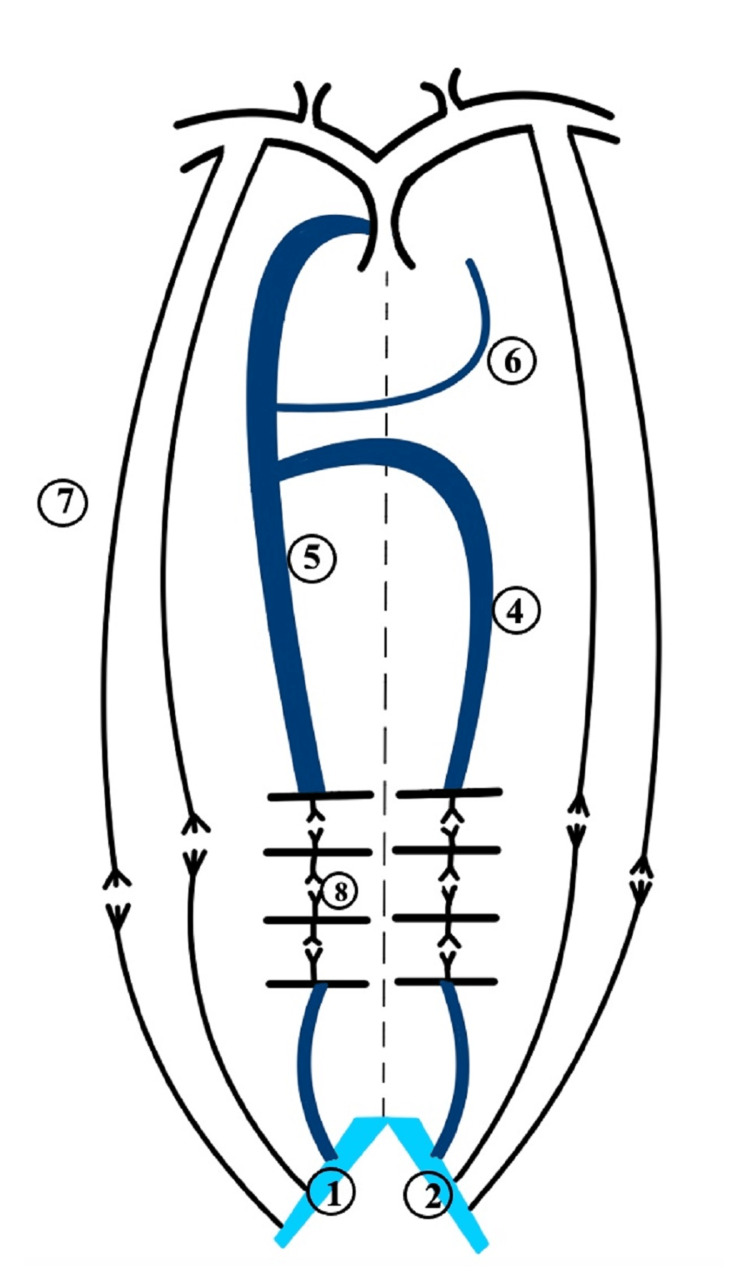
Proposed embryology of agenesis of inferior vena cava and enlarged collateral veins Right common iliac vein (1); left common iliac vein (2); inferior vena cava (3); hemiazygos vein (4); azygos vein (5); accessory hemiazygos vein (6); abdominal wall collaterals (7); lumbar and paralumbar veins (8)

Embryologically, the IVC is usually derived from parts of the three vessels during six to eight weeks of the embryonic period (Figure 3) [14]. The right supracardinal vein contributes to the infrarenal segment of the IVC, and the renal part develops from the supra-sub cardinal anastomosis [14]. The right subcardinal vein forms the suprarenal component, and the hepatic segment develops from the vitelline vein [14]. The azygos and the hemiazygos veins are formed from parts of the supracardinal veins (Figure 4) [14]. The remaining parts of the embryological venules disappear. Anomalies of the IVC due to failed regression or persistence of embryological channels occur in less than 1% of cases [8]. In this case, IVCA was likely related to the defective development of the three IVC segments [4,15]. An error in the union of the right subcardinal vein with the hepatic sinusoids possibly led to the diversion of blood from the lower extremities to the azygos system. Hence, these vessels got secondarily enlarged [14,15]. Since the right and left supracardinal veins connect the two posterior cardinal veins during the embryonic period, the connection persisted when IVC was not formed. Additionally, the lumbar veins, paralumbar veins, and collateral pathways in the abdominal walls carried blood from the lower extremities, abdomen, and pelvis into the superior vena cava (Figure 5).

The other anatomical variations of the IVC reported include drainage of IVC into the left atrium, pulmonary venous drainage into the IVC, absence of suprarenal or infrarenal IVC, duplication of IVC, left IVC, retro aortic or circum-aortic renal veins, and retrocaval ureter [16]. Defective development of the IVC in the embryo can be accompanied by anomalies involving other organs [4]. These defects are mainly related to the spleen, liver, heart, and lungs [4]. The commonly associated cardiac defects are dextrocardia, atrial septal defects, atrioventricular canal, or pulmonary stenosis [15]. In this case, the left kidney was atrophic, and the right kidney was hypertrophic. The left renal atrophy is probably due to impaired venous drainage related to IVCA. A similar finding was noted in a 16-year-old female with IVCA [17]. However, the DVT was bilateral, and the authors called this condition kidney, IVC, leg thrombosis (KILT) syndrome [17]. 

The IVCA has been reported in patients with DVT with no history of risk factors for venous thromboembolism or clotting diathesis [4]. This patient had DVT likely due to venous stasis that resulted from IVCA, as other predisposing factors were ruled out. Another patient, a 39-year-old male with no risk factors, was diagnosed with DVT due to the absence of the infrarenal portion of the IVC [13]. Deep vein thrombosis can occur due to genetic and acquired causes, which increase the risk factors for patients with anomalies in the IVC [15]. Although extensive collaterals replace the venous drainage of an absent IVC, the patients may still have chronic venous hypertension leading to venous stasis and, thereby, thrombosis [9]. It is recommended that patients with IVC anomaly should be screened for the thrombophilic disorder as 7 out of 9 patients with IVC anomaly and DVT were found to have a positive thrombophilic screen [18]. Especially in young patients with no previous risk factors, IVCA is a significant risk factor for unprovoked DVT in the lower extremities, which may require lifelong anticoagulation [19]. 

## Conclusions

Inferior vena cava agenesis is a significant but highly underrecognized cause of unprovoked DVT of the lower extremities in the young population without additional risk factors. Hence, a complete diagnostic evaluation is necessary, which includes imaging for vascular anomalies besides the thrombophilic screen. Given the condition’s rarity, there is no clear evidence in the literature for guidelines-based management. Treatment is mainly conservative and focused on preventing venous stasis, clot formation, and recurrence, which could be achieved by lifelong anticoagulation in addition to compression stockings and leg elevation. The surgical option could be possible depending on the severity of symptoms and response to medical therapy.
